# Metabolic and Transcriptomic Basis of Quality Divergence in Onions (*Allium cepa* L.) with Different Bulb Colors

**DOI:** 10.3390/plants15131949

**Published:** 2026-06-24

**Authors:** Chenghai Shan, Chenlu Zhang, Xuena Yu, Wenyou Zhang, Lin Yang, Xuan Dong, Deping Wu, Bo Sun

**Affiliations:** 1 College of Agricultural Sciences, Xichang University, Xichang 615013, China; zhangwenyou2027@163.com (W.Z.); yanglinlinxc@126.com (L.Y.); xcc20240009@xcc.edu.cn (X.D.); wudeping2026@163.com (D.W.); 2 College of Horticulture, Sichuan Agricultural University, Chengdu 611130, China; 2024105007@stu.sicau.edu.cn (C.Z.); 80251@sicau.edu.cn (X.Y.)

**Keywords:** onion, metabolome, volatile compounds, antioxidant capacity, flavor, transcriptome

## Abstract

Four onion (*Allium cepa* L.) cultivars with different bulb colors (yellow Y14, red R12, red R10 and white W3) were characterized for phenotypic, metabolic, volatile, antioxidant, flavor and transcriptomic variations, to unravel key metabolites and molecular mechanisms responsible for quality differentiation. Y14 possessed the maximum bulb weight (535.34 g) and diameter (13.10 cm), along with the highest total phenolic content (3.10 mg·g^−1^), showing superior yield and antioxidant properties. In R12, upregulated expression of carotenoid biosynthetic genes enhanced carotenoid accumulation, resulting in vivid bulb color. R10 had high total phenols (3.89 mg·g^−1^) and the richest sweet-associated volatiles, featuring strong antioxidant activity and distinct sweetness. By contrast, W3 exhibited moderate flavor but inferior performance in yield, antioxidant capacity, pigment and volatile levels relative to the other three cultivars. Comprehensive assessment indicated that Y14 serves as an excellent processing material, R12 and R10 are ideal for fresh consumption and functional food development, and W3 is well-suited for raw salads. This study lays a theoretical foundation for onion quality improvement, targeted breeding and efficient utilization.

## 1. Introduction

Onion (*Allium cepa* L.) is a biennial herb belonging to the family Amaryllidaceae, genus *Allium*, and is one of the most widely cultivated and consumed vegetable crops worldwide [1]. It is rich in organosulfur compounds, flavonoids, phenolic acids, anthocyanins, ascorbic acid, and carotenoids, which confer strong antioxidant, anti-inflammatory, antimicrobial, cardiovascular-protective, and immune-modulating activities, making onion a typical medicinal and edible crop [2,3,4]. With increasing demand for healthy diets, nutritional quality, functional components, flavor characteristics, and processing suitability have become core indicators for variety evaluation, market selection, and industrial development of onion [5].

Onion cultivars are commonly classified into red, yellow, and white types based on bulb skin color, which show significant divergence in morphological traits, phytochemical composition, antioxidant capacity, flavor profiles, and end-use suitability [6,7]. Yellow onions feature high yield, good storability, and suitable pungency, and are widely used in dehydration, freezing, and other processing industries [8,9]. Red onions are characterized by high contents of anthocyanins and total phenolics, superior antioxidant activity, and attractive appearance, making them ideal for fresh consumption and functional food development [6]. White onions have tender flesh, bright color, and mild flavor, and are preferred for fresh salads and light cooking [10]. Previous studies confirmed that bulb color is closely associated with the accumulation of secondary metabolites and volatile compounds, which further determine consumer preference and industrial utilization [11,12,13]. However, systematic integration of phenotypic, metabolic, transcriptomic, and flavor differences among color-divergent onion varieties remains limited, hindering precise breeding for fresh-market and processing-specific cultivars.

Integrated metabolomic and transcriptomic analysis has become a powerful strategy to dissect the molecular basis of quality formation in horticultural crops [14,15,16,17], enabling identification of key differential metabolites, regulatory pathways, and functional genes related to target traits [18,19]. In onion research, omics approaches have been applied to reveal regulatory mechanisms of anthocyanin biosynthesis, flavonoid accumulation, flavor metabolism, and stress responses [20]. Nevertheless, comprehensive multi-omics studies focusing on quality differentiation and breeding implications among yellow, red, and white onions for fresh and processing uses are still scarce, especially regarding the coordinated regulation of color brightness, antioxidant capacity, volatile profiles, and taste characteristics.

In this study, four representative onion cultivars, including one yellow cultivar (Y14), two red cultivars (R10 and R12), and one white cultivar (W3), were used to systematically compare the differences in key traits such as phenotypic characteristics, metabolite profiles, and flavor quality. Furthermore, transcriptomic analysis was employed to elucidate the molecular mechanisms underlying the observed quality differentiation. The results of this study will provide a theoretical basis for the efficient utilization of onion cultivars with different bulb colors and offer elite germplasm for the genetic improvement of onion quality.

## 2. Results

### 2.1. Yellow Onion Had the Largest Single Bulb Weight, and Red Onions Exhibited More Attractive Appearance

**Figure 1:**
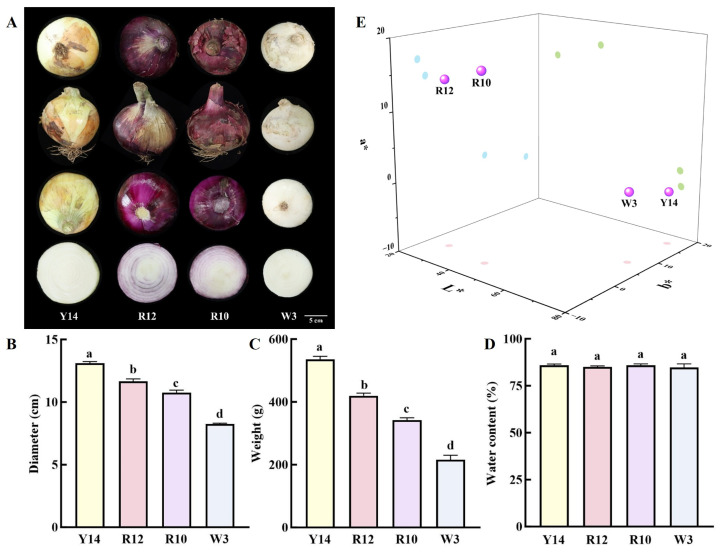
Phenotype (**A**), color parameters (**B**), bulb diameter (**C**), single bulb weight (**D**) and moisture content (**E**) of four onion (*Allium cepa* L.) cultivars. Values represent the mean ± SD of three biological replicates. The same letter in the same histogram indicates that there is no significant difference between the values tested by least significant difference (LSD) (*p* < 0.05).

Phenotypic observation showed that the bulb of yellow onion was the largest among the four cultivars. The maximum diameter was 13.10 cm, which was 1.12-, 1.22-, and 1.59-fold that of R12 (11.65 cm), R10 (10.75 cm), and W3 (8.25 cm), respectively. The single bulb weight was 535.34 g, which was 1.28-, 1.56-, and 2.48-fold that of R12 (419.80 g), R10 (342.48 g), and W3 (215.51 g), respectively (Figure 1A–C). The moisture content was similar among different onion cultivars, at approximately 85% (Figure 1D).

Color difference analysis revealed that three types of onions with different colors could be distinguished by three parameters: L*, a*, and b*. Yellow and white cultivars showed brighter appearance, consistent with their significantly higher L* values (73.75 and 73.20) than those of red cultivars (27.68 and 44.13). Red cultivars presented attractive reddish-purple color, and R10 exhibited deeper purple. Accordingly, the a* values of red onions (14.03 and 16.50) were significantly higher than those of yellow (−2.24) and white (0.07) onions, with R10 showing the maximum a* value. In addition, the b* values of red onions were negative (−3.98 and −5.30), much lower than those of yellow (16.88) and white (7.54) onions. These results indicate that yellow onion shows a high-yield characteristic, while red onions possess more attractive bulb color.

### 2.2. Organic Acids and Derivatives as Well as Benzenoids Were the Predominant Specific Differential Metabolites Among Four Onion Cultivars with Different Bulb Colors

**Figure 2:**
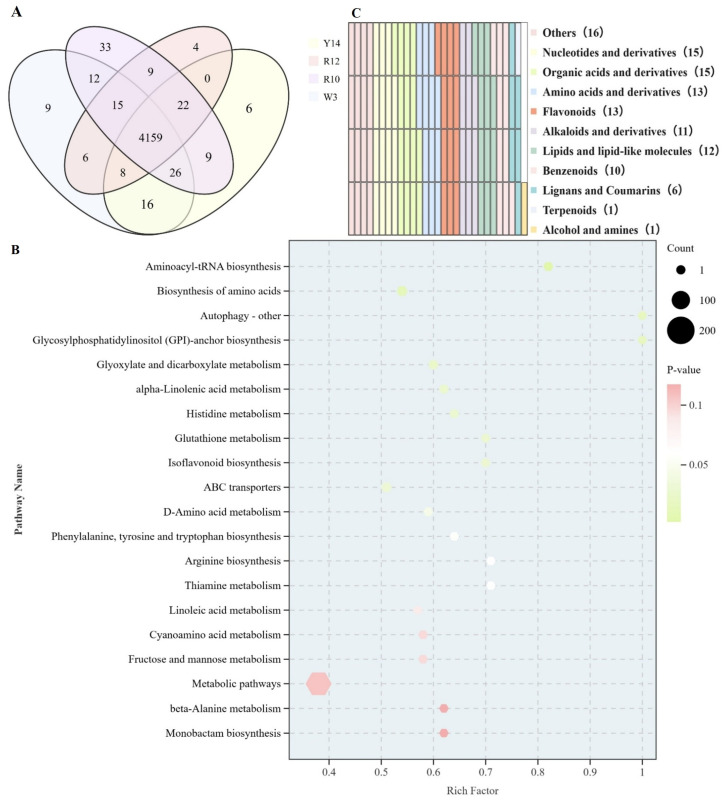
Metabolite profiling of four onion cultivars. (**A**) Venn diagram of all metabolites; (**B**) Classification of metabolites; (**C**) KEGG enrichment analysis of differential metabolites.

A total of 4159 common metabolites were identified across the four onion cultivars via untargeted metabolomics (Figure 2A). These metabolites were classified into 21 categories. Organic acids and derivatives accounted for the largest proportion (24.29%), followed by benzenoids (11.64%), lipids and lipid-like molecules (9.79%), and flavonoids (9.67%). The relative content of the remaining categories was all less than 5% (Figure S1A). Principal component analysis (PCA) showed that the first principal component (PC1) and the second principal component (PC2) explained 29.36% and 22.31% of the total variance, respectively, with a cumulative contribution rate of 51.67%, which well reflected the overall metabolomic differences among samples. Y14 and W3 shared similar metabolic profiles, which were distinctly different from those of R12 and R10, suggesting that yellow and white onions had analogous metabolite compositions, whereas obvious metabolic divergence existed between yellow/white and red onions. Additionally, R12 and R10 were clearly separated along the PC2 axis, indicating significant metabolic variation between the two red cultivars (Figure S1B).

In total, 1266 differential metabolites (DAMs) were screened out, among which 252 were annotated to 96 KEGG pathways. The metabolic pathways contained the largest number of DAMs (184), and 113 of them were assigned to Biosynthesis of secondary metabolites (Figure 2B). Among these 113 secondary DAMs, nucleotides and derivatives, as well as organic acids and derivatives were the most abundant (15 metabolites for each category), followed by amino acids and derivatives and flavonoids (13 metabolites for each category). Terpenoids, alcohols and amines had the lowest abundance (only 1 metabolite for each category) (Figure 2C). These results demonstrated that phenolic compounds and flavonoids (secondary metabolites) were the major functional substances differentiating the four onion cultivars.

**Figure 3:**
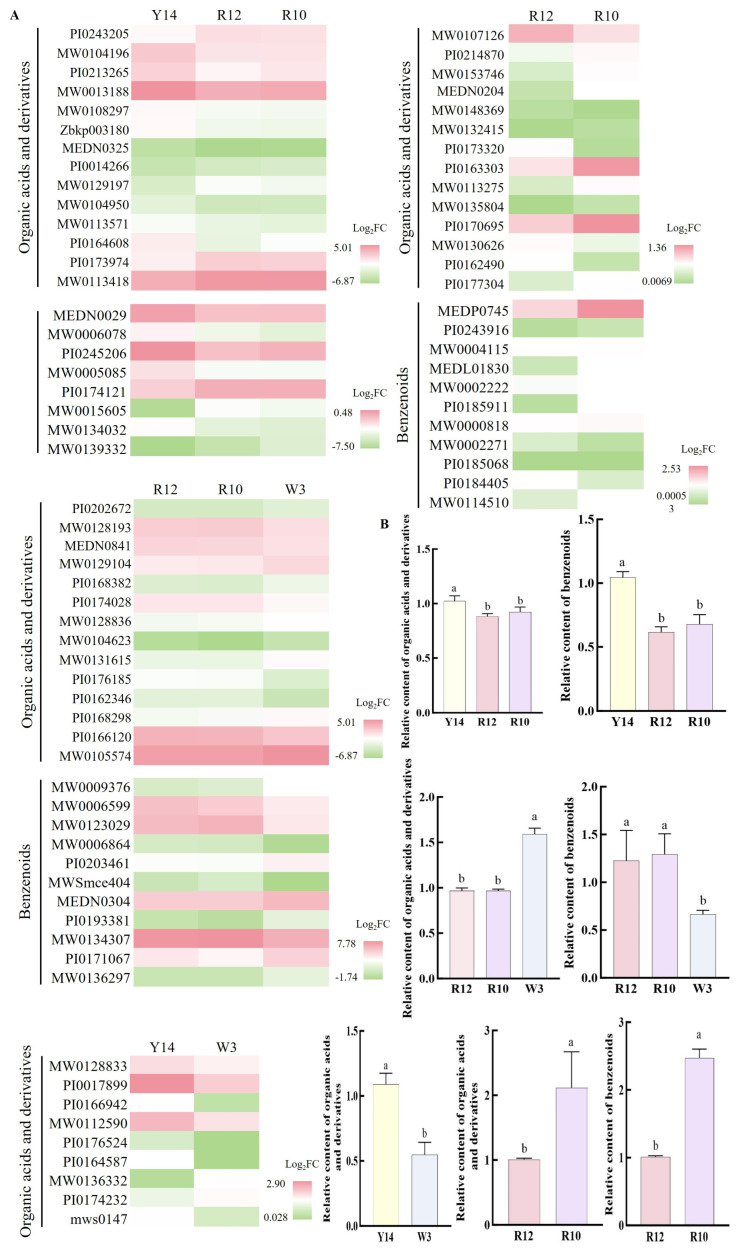
Difference analysis of metabolites in four onion varieties. (**A**) Heatmap of cultivar-specific differential metabolites; (**B**) Relative contents of specific organic acids and derivatives as well as benzenoids in different comparison groups. Bars are means ± SD of three biological replicates. The same letter in the same histogram indicates that there is no significant difference between the values tested by least significant difference (LSD) (*p* < 0.05).

Further analysis on cultivar-specific differential metabolites showed that organic acids and derivatives were the most abundant specific DAMs between yellow/white onions and red onions (14 metabolites for each comparison group), followed by benzenoids (8 and 11 metabolites, respectively) (Figure S1C). The contents of these two categories in red onions were significantly lower than those in yellow onions: organic acids and derivatives in red onions were 85% and 90% of those in yellow onions, while benzenoids were 59% and 65% of the levels in yellow onions. The content of organic acids and derivatives in red onions was approximately 60% of that in white onions, whereas the content of benzenoids in red onions was nearly twice that in white onions. Benzenoids (9 metabolites) were the main specific DAMs between yellow and white onions, and their content in yellow onions was about twice that in white onions. Between the two red cultivars, organic acids and derivatives (14 metabolites) and benzenoids (11 metabolites) were also the dominant specific DAMs, and both categories accumulated more in R10, with contents 1.45-fold and 2.45-fold higher than those in R12, respectively (Figure 3 and Figure S1D). In conclusion, organic acids and derivatives and benzenoids were the primary specific differential metabolites among the four onion cultivars. Yellow onions accumulated the highest levels of these two substances. Red onions had the lowest content of organic acids and derivatives, and white onions had the lowest content of benzenoids.

### 2.3. Red Onion Cultivar R10 Exhibited the Strongest Antioxidant Capacity

**Figure 4:**
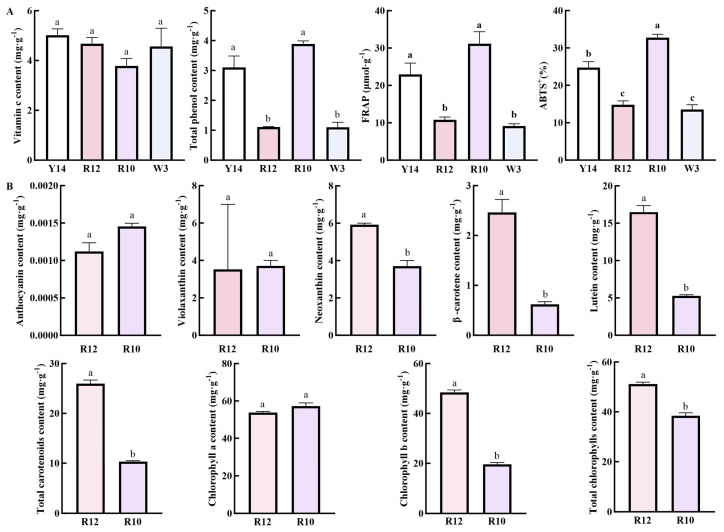
Antioxidant capacity (**A**) and pigments content (**B**) of four onion cultivars. Bars are means ± SD of three biological replicates. The same letter in the same histogram indicates that there is no significant difference between the values tested by least significant difference (LSD) (*p* < 0.05).

Ascorbic acid belongs to organic acids and derivatives, and phenolic compounds are typical benzenoids. Both are important indicators for evaluating onion quality and closely associated with antioxidant activity. In this study, the contents of ascorbic acid and total phenols were determined in four cultivars. No significant difference in ascorbic acid content was detected among all cultivars. The total phenolic contents of Y14 and R10 were approximately 3-fold higher than those of R12 and W3. Consistently, R10 presented the highest antioxidant activities evaluated by FRAP and ABTS assays, followed by Y14, and both were significantly higher than R12 and W3. These results confirmed that R10 had the strongest antioxidant capacity, while R12 and W3 showed relatively weak antioxidant performance (Figure 4A). exerting crucial effects on the sensory and nutritional quality of onions. The two red onion cultivars exhibited comparable anthocyanin contents; nevertheless, R12 displayed significantly higher lightness values than R10 (Figure 1E). Accordingly, we hypothesize that carotenoids and chlorophyll also participate in color development in red onions. Quantification assays revealed that the contents of β-carotene and lutein in R12 were more than threefold those in R10, resulting in a total carotenoid content over 2.5 times higher in R12 relative to R10. Meanwhile, chlorophyll concentrations showed no significant difference between R12 and R10, which ultimately conferred a brighter bulb color to R12 (Figure 4B).

### 2.4. Red Onion Cultivar R10 Accumulated the Highest Content of Volatile Metabolites

**Figure 5:**
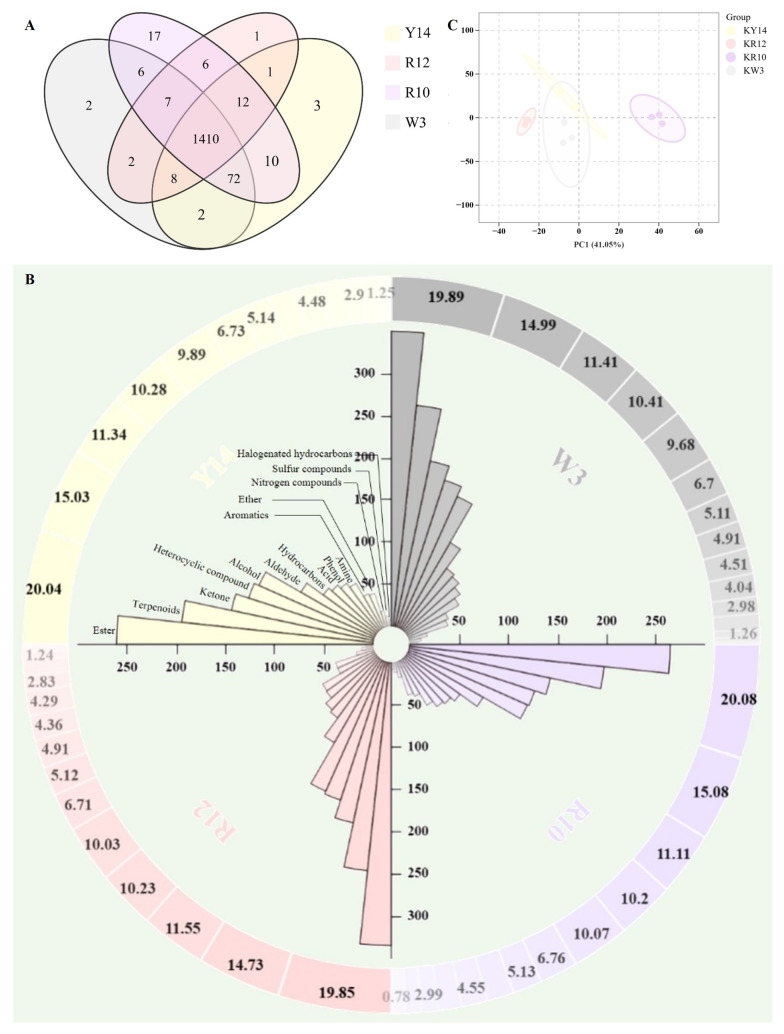
Analysis of volatile metabolites in four onion cultivars. (**A**) Venn diagram of volatile metabolites; (**B**) Classification of volatile metabolites; (**C**) PCA plot of volatile differential metabolites.

The composition and content of volatile metabolites are critical factors determining vegetable flavor. To clarify the flavor differences among the four onion cultivars, gas chromatography-mass spectrometry (GC-MS) was applied to profile their volatile metabolites. A total of 1410 common volatile compounds were detected in all cultivars (Figure 5A), which were divided into 15 categories. Esters were the most abundant group, accounting for approximately 20% of total volatiles, followed by terpenoids (around 15%). Ketones, heterocyclic compounds and alcohols each occupied about 10%, while halogenated hydrocarbons were the least abundant (only ~1%) (Figure 5B). PCA of volatile metabolites showed that PC1 and PC2 explained 41.05% and 20.24% of the total variance, with a cumulative contribution rate of 61.29%, which fully reflected the overall variation of volatile compounds across samples. Y14 and W3 were closely distributed along the PC1 axis, indicating high similarity in their overall volatile profiles. However, they were clearly separated on the PC2 axis, suggesting specific differences in partial characteristic volatile components. R12 was concentrated on the negative side of PC1, while R10 gathered on the positive side of PC1. The two red cultivars were distant from yellow and white onions, demonstrating remarkable divergence in volatile compositions. These results indicated obvious differentiation in volatile metabolic profiles among onions with different bulb colors (Figure 5C).

**Figure 6:**
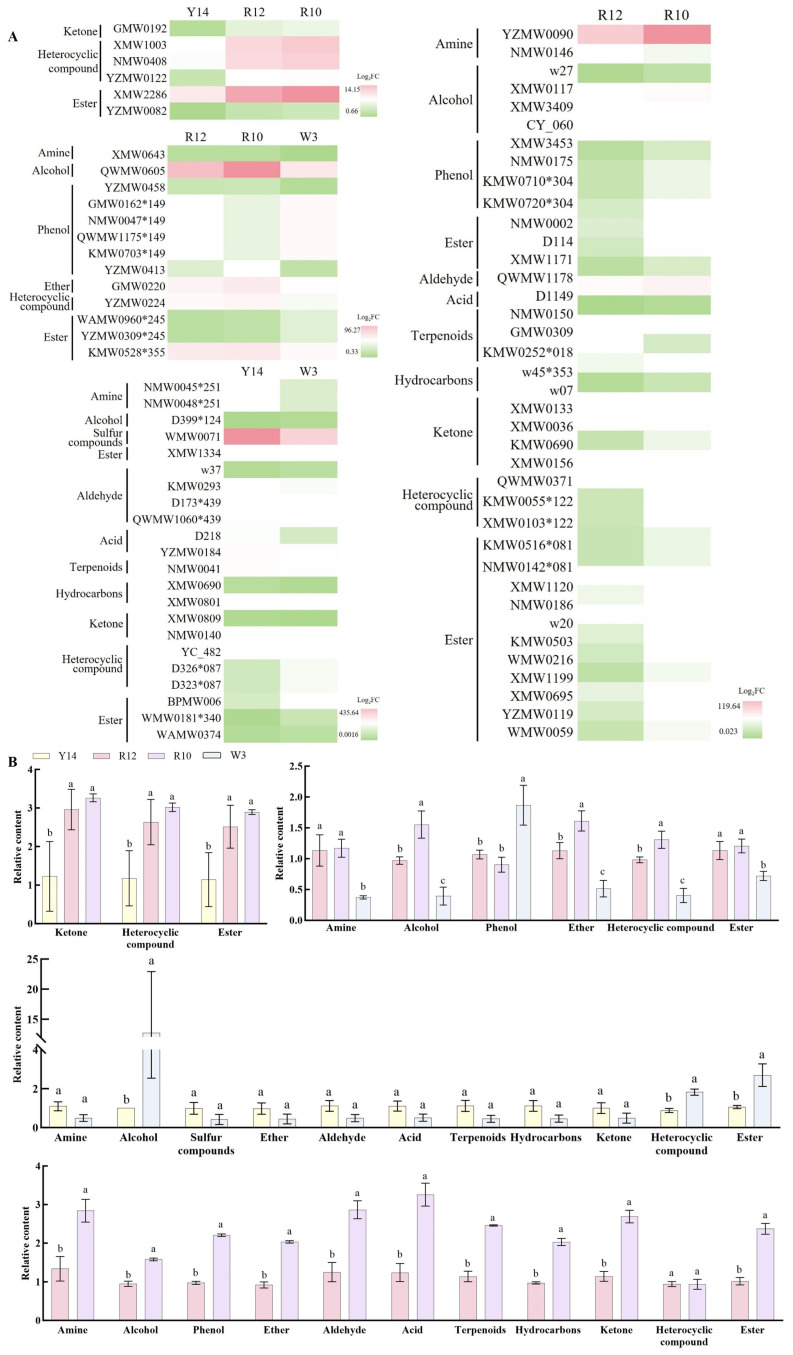
Difference analysis of volatile metabolites in four onion varieties. (**A**) Heatmap of cultivar-specific volatile differential metabolites; (**B**) Relative contents of specific volatile metabolites in different comparison groups. Bars are means ± SD of three biological replicates. The same letter in the same histogram indicates that there is no significant difference between the values tested by least significant difference (LSD) (*p* < 0.05).

Overall, 465 volatile differential metabolites were identified, and 38 of them were annotated to KEGG pathways. The Metabolic pathways contained the maximum number of volatile DAMs (28), including 14 metabolites assigned to biosynthesis of secondary metabolites (Figure S2A), which was consistent with the enrichment pattern of non-volatile DAMs. The 38 volatile DAMs were classified into 9 categories. Terpenoids were the most abundant (9 metabolites), followed by acids (8) and alcohols (7). The remaining six categories contained fewer than 5 metabolites, with esters being the least abundant (only 1 metabolite) (Figure S2B). This result suggested that terpenoids were the main volatile substances driving flavor differences among the four onion cultivars. Analysis of cultivar-specific volatile DAMs showed the following results (Figure S2C): Six specific volatile DAMs (four categories) were identified between yellow and red onions, and all of them accumulated significantly more in red onions. Thirteen specific volatile DAMs (six categories) were detected between white and red onions; phenols were more abundant in white onions, whereas the other five categories were significantly enriched in red onions. Twenty-two specific volatile DAMs (eleven categories) existed between yellow and white onions. Only alcohols showed a remarkable difference, with the content in white onions being 12.73-fold that in yellow onions, and no significant difference was observed for the rest categories. Thirty-eight specific volatile DAMs (eleven categories) were found between the two red cultivars. Except for heterocyclic compounds with no significant variation, the other ten categories had significantly higher contents in R10 than in R12 (Figure 6). In summary, red onions had higher total contents of volatile metabolites than yellow and white onions, and R10 had the highest level of volatile compounds.

### 2.5. Red Onion Cultivar R10 Possessed the Most Prominent Sweet Flavor

**Figure 7:**
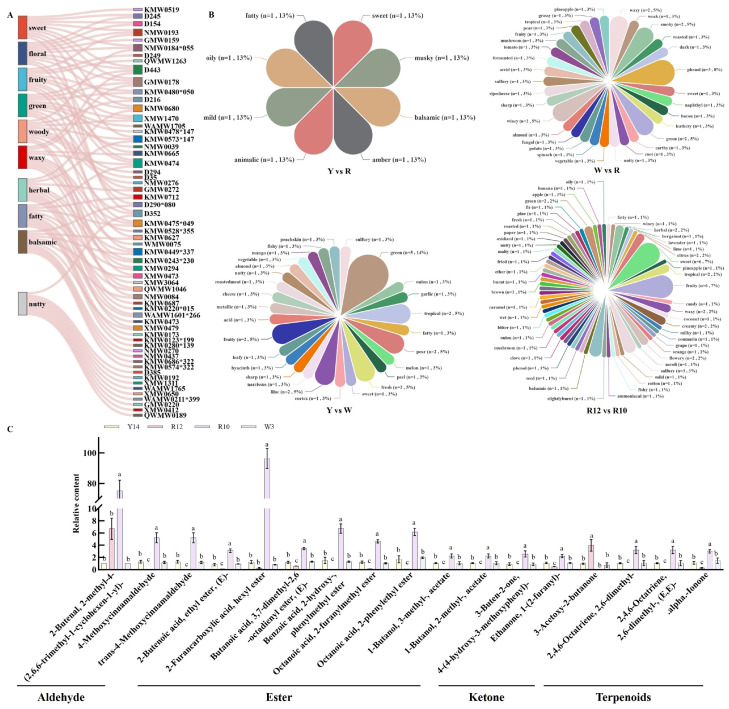
Flavor characteristic analysis of four onion cultivars. (**A**) Sankey diagram of flavor-related differential metabolites; (**B**) Classification of cultivar-specific flavor-related metabolites in different comparison groups; (**C**) Relative contents of specific flavor-related metabolites in different comparison groups. Bars are means ± SD of three biological replicates. The same letter in the same histogram indicates that there is no significant difference between the values tested by least significant difference (LSD) (*p* < 0.05).

Volatile metabolites are the key determinants of vegetable flavor, so we further analyzed the flavor variations among the four onion cultivars. A total of 338 volatile DAMs were involved in flavor regulation. The compounds related to sweet flavor were the most numerous (61 metabolites), followed by fruity, green, floral, woody, fatty, herbal and waxy flavors, each regulated by more than 20 metabolites (Figure S2D). Among all flavor types, sweet flavor showed the most significant variation across cultivars, followed by floral, fruity, green, woody, waxy, herbal and fatty flavors in sequence (Figure 7A).

Analysis of cultivar-specific flavor differences revealed that yellow and red onions had the smallest flavor divergence, with differences in only 8 flavor types, and each flavor was regulated by a single volatile DAM. By contrast, the two red cultivars exhibited the largest flavor variation, with significant differences in 67 flavor types modulated by 20 volatile DAMs. Metabolites related to sweet and fruity flavors were the most abundant (6 metabolites for each). Notably, the flavor difference between yellow and white onions was mainly reflected in green flavor, and no obvious difference was detected in sweet flavor (Figure 7B). Collectively, sweet flavor was the core index distinguishing the four onion cultivars. The flavor variation between the two red cultivars was greater than that between onions of different colors. Quantitative analysis of sweet-related volatile DAMs showed that all compounds except 3-Acetoxy-2-butanone (QWMW1069) had the highest contents in R10. Apart from 2-Butenal, 2-methyl-4-(2,6,6-trimethyl-1-cyclohexen-1-yl)- (D352) and 3-Acetoxy-2-butanone, all other sweet-related metabolites presented the lowest levels in R12. No significant difference in sweet-related compounds was found between yellow and white onions. These findings indicated an extreme divergence in sweet metabolite accumulation between the two red cultivars: R12 was deficient in sweet substances while R10 was rich in sweet compounds, which accounted for the distinct sweet flavor between red onions and yellow/white onions (Figure 7C).

### 2.6. Red Onion R10 Had the Strongest Phenylpropanoid Biosynthesis Capability, While R10 and R12 Exhibited Superior Anthocyanin and Carotenoid Biosynthesis Capacities, Respectively

**Figure 8:**
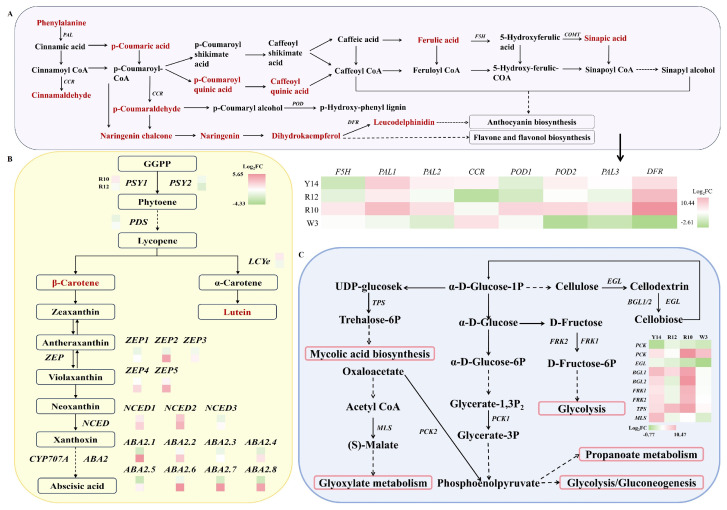
Metabolic pathways and expression profiles of key DEGs involved in phenylpropanoid (**A**), carotenoid (**B**) and sugar metabolism (**C**) in four onion cultivars. Solid arrows correspond to one-step reactions, with the exception of the solid arrow connecting the pathway scheme to the gene expression heatmap. Dashed arrows denote metabolic reactions with omitted intermediate steps.

RNA-seq was performed to explore the molecular mechanisms underlying the differences in bulb color, metabolites and flavor among the four onion cultivars at the transcriptional level. Eight differentially expressed genes (DEGs) were identified in the phenylpropanoid metabolism pathway. Except for the *CCR* gene, the other seven DEGs had the highest expression levels in R10, which explained the higher total phenolic content of R10. Moreover, the expression of anthocyanin biosynthesis-related genes such as *DFR* was significantly higher in red onions than in yellow and white onions, leading to stronger anthocyanin synthesis and greater anthocyanin accumulation in red onions (Figure 8A). In the carotenoid metabolism pathway, the expression levels of *PSY*, *PDS* and *LCYe* were significantly higher in R12 than in R10, while the expression of *ZEP*, *NCED* and *ABA* synthesis-related genes was markedly lower in R12. This transcriptional pattern indicated that the total flux of carotenoid metabolism was enhanced and the lutein biosynthesis branch was activated in R12, which was consistent with the higher contents of β-carotene, lutein and total carotenoids in R12 (Figure 8B). For sugar metabolism pathways, *EGL* and *MLS* showed the highest expression in yellow onions, whereas all other DEGs were most highly expressed in R10. This expression profile was consistent with the prominent sweet flavor of R10 (Figure 8C).

## 3. Discussion

Onion bulb color is a typical intuitive trait closely linked to phytochemical composition, antioxidant potential, flavor profile, and commercial value. In this study, we integrated phenotypic observation, metabolomics, volatile component analysis, antioxidant detection and transcriptome sequencing to systematically explore quality variation across four onion cultivars with different bulb colors. The multi-omics datasets revealed clear divergence in morphological characteristics, pigment metabolism, secondary metabolites, flavor substances and gene expression patterns. The outcomes deepen our understanding of the metabolic and molecular mechanisms underlying quality differentiation in colored onions, and offer theoretical references for germplasm utilization and targeted breeding.

Bulb size and appearance quality directly determine the commodity and market value of onions [21]. Consistent with previous reports, yellow onion generally presents superior yield performance. In the present study, Y14 showed the largest bulb size among all test materials. Distinct color characteristics were observed between the two red onion cultivars: R12 displayed a brighter bulb appearance, while R10 exhibited a deeper purple hue. Further pigment analysis confirmed that apart from anthocyanins, carotenoids including β-carotene and lutein also play a vital role in regulating bulb brightness in red onions. This finding complements the existing knowledge of onion color formation, which has long focused primarily on anthocyanin metabolism [22,23].

Secondary metabolites such as phenolic compounds and flavonoids are well-recognized major contributors to the antioxidant capacity of Allium crops [24,25,26]. Multiple studies have verified that red onions usually contain higher levels of total phenols and possess stronger antioxidant activity than white onions [6,27], which is consistent with our research results. In this study, phenolic substances varied obviously across cultivars, which consequently led to divergent antioxidant activity. Overall, red onion R10 and yellow onion Y14 possessed stronger antioxidant properties, whereas R12 and white W3 were relatively weaker in this regard. Ascorbic acid, another common antioxidant component, showed no obvious differences among the four cultivars, indicating that phenolics rather than ascorbic acid dominated the antioxidant capacity of these onion materials. Differing from the conclusion of He et al. (2026) that purple onions have the highest flavonoid content [6], our work found that high total phenols also existed in high-yield yellow onion Y14, which reflects the combined influence of genotype and germplasm differences on secondary metabolite accumulation. Such variation in antioxidant performance is consistent with the metabolic divergence observed across different bulb color groups.

Flavor is a core consumer-oriented quality trait determined by volatile metabolites [11]. Previous volatile metabolomics studies on colored onions indicated that esters, terpenoids and sulfur-containing compounds are the dominant volatile components of onion bulbs [4,9,10], which is highly consistent with the component classification obtained in this research. Noticeably, red onions accumulated more volatile compounds than yellow and white onions, and terpenoids were the key substances driving flavor differences among cultivars. Sweet flavor represented the most variable sensory characteristic in this study. Intriguingly, the two red cultivars exhibited larger flavor differences than onions of different colors. Meanwhile, the distinct flavor differences observed between the two red cultivars also reflect the intraspecific variation in flavor traits among red onion germplasms. These metabolic features provide important clues for the selective breeding of fresh-eating and processing-type onion varieties.

It should be emphasized that bulb color alone cannot define the processing suitability of onion cultivars. The evaluation of processing-oriented varieties requires a comprehensive consideration of multiple indicators including yield, bulb texture, flavor stability during processing, and field disease resistance. Combined with the phenotypic and quality data obtained in this study, Y14 with high yield and relatively mild and stable flavor is more adaptable to industrial processing. In contrast, R10 and R12 with rich volatile components and distinct taste are more suitable for fresh consumption and functional food production, and their relatively low yield also limits their large-scale processing application. White onion W3 has tender flesh and mild flavor for raw salad and light cooking, but its inferior yield restricts its promotion as a major processing material [10].

Transcriptome analysis further revealed potential regulatory mechanisms associated with metabolic and phenotypic divergence among the four onion cultivars. In the phenylpropanoid pathway, seven out of eight DEGs exhibited relatively higher expression in R10, which was correlated with the increased biosynthesis of total phenols and anthocyanins as well as improved antioxidant capacity. The expression of *DFR* and other structural genes involved in anthocyanin biosynthesis was markedly elevated in red onions. This expression pattern is in line with previous studies, which demonstrated that an activated anthocyanin pathway contributes to the formation of purple bulb color [28,29]. In the carotenoid pathway, R12 presented higher transcript levels of *PSY*, *PDS* and *LCYe*, accompanied by lower expression of *ZEP*, *NCED* and ABA synthesis-related genes. Such expression changes may redirect metabolic flux toward carotenoid and lutein accumulation, which likely accounts for its brighter bulb color. In sugar metabolism pathways, most DEGs showed the highest expression in R10, matching its prominent sweet flavor trait. Collectively, the coordinated changes in gene expression within phenylpropanoid, carotenoid, and sugar metabolic pathways are closely associated with the differentiation of bulb color, antioxidant activity, and flavor in onions. Combined transcriptomic, metabolomic and phenotypic data jointly support these correlations. Further functional verification of candidate genes will be carried out in our future work.

## 4. Materials and Methods

### 4.1. Plant Materials

Four onion cultivars with three bulb colors, namely yellow (Y14), red (R12, R10), and white (W3), independently bred by the Onion Breeding Team of Xichang University, were used as experimental materials. Bulbs were harvested at full ripeness, corresponding to 180 d, 175 d, 215 d and 205 d after transplanting for each cultivar, respectively. For phenotypic analysis, High-performance liquid chromatography/Gas Chromatography-Mass Spectrometry (HPLC/GC-MS) detection and transcriptome sequencing, three representative bulbs per cultivar were selected from a large field population. These samples were randomly chosen to reflect the overall growth status and phenotypic characteristics of the whole population, and served as independent biological replicates in subsequent experiments.

### 4.2. Measurement of Maximum Bulb Diameter

The transverse maximum diameter of each onion bulb (the widest point perpendicular to the pseudostem) was precisely measured using a vernier caliper. Three biological replicates were performed, and the average value was defined as the maximum diameter of the corresponding cultivar.

### 4.3. Determination of Relative Water Content

The fresh weight (W_1_) of each intact onion bulb was first recorded. Afterwards, each bulb was cut into small pieces to accelerate water removal. and the fragmented tissues were then oven-dried at 65 °C for 4 h until constant weight was achieved. After drying, all dried tissues were collected and weighed as the dry weight of the corresponding whole bulb (W_2_). The relative water content was calculated using the following formula: Relative water content (%) = (W_1_ − W_2_) × 100/W_1_.

### 4.4. Color Difference Analysis

Color difference analysis was performed according to the method established by Zhang et al. [30]. An NR110 colorimeter (3nh, Shenzhen, China) was employed to analyze the bulb color of onions. Three random positions on the outer fleshy scale leaves of each onion bulb were selected to record L*, a*, and b* values. According to the manufacturer’s instructions: −L* indicates blackness, +L* indicates whiteness; −a* indicates greenness, +a* indicates redness; −b* indicates blueness, and +b* indicates yellowness.

### 4.5. Non-Targeted Metabolomic Detection

Non-targeted metabolomics of onion bulbs was performed using an UPLC-MS/MS system (Shimadzu LC-30A (Kyoto, Japan) coupled with SCIEX TripleTOF 6600+ (Foster City, CA, USA)). Freeze-dried bulb powder (30 mg) was extracted with 1500 μL of pre-cooled 70% methanol, and separation was carried out on a Waters ACQUITY UPLC HSS T3 column with 0.1% formic acid in water and acetonitrile as mobile phases. Mass spectrometry data were acquired in both positive and negative ESI modes with a mass range of 50–1250 Da. Raw data were processed using ProteoWizard (Version 3.0.7414) and XCMS (Version 3) for peak extraction, alignment, and correction; metabolites with identification score > 0.5 and CV < 0.5 in QC samples were kept for analysis. Differential metabolites were identified based on VIP > 1 from OPLS-DA models, combined with |log_2_FC| ≥ 1 (two groups) or ANOVA *p* < 0.05 (multiple groups).

### 4.6. Volatile Metabolite Profiling (GC-MS)

Volatile organic compounds (VOCs) in onion bulbs were extracted by headspace solid-phase microextraction (HS-SPME) with a 120 μm DVB/CWR/PDMS fiber at 60 °C for 15 min and analyzed using an Agilent 8890 GC (Agilent Technologies, Santa Clara, CA, USA) coupled with a 7000D mass spectrometer equipped with a DB-5MS column (30 m × 0.25 mm × 0.25 μm). Helium was used as the carrier gas at 1.2 mL/min, and the oven temperature was programmed from 40 °C to 280 °C. Mass spectra were acquired in electron impact (EI) mode at 70 eV using selected ion monitoring (SIM). Raw data were processed by MassHunter software (Version B.08.00), and only metabolites with CV < 0.5 in quality control samples were retained. Differential volatile metabolites were screened by variable importance in projection (VIP > 1) combined with fold change (|log_2_FC| ≥ 1) or ANOVA (*p* < 0.05) using OPLS-DA models. Relative odor activity values (rOAV) were calculated to evaluate key aroma-contributing metabolites.

### 4.7. Transcriptome Sequencing and Analysis

Total RNA was extracted from onion bulbs, and mRNA was subsequently purified for cDNA library construction. All transcriptome sequencing experiments were performed with three independent biological replicates for each cultivar to ensure data reliability. After quality control, the qualified libraries were sequenced on the Illumina high-throughput sequencing platform. Raw sequencing reads were filtered using fastp software (Version 0.23.2) to remove low-quality reads and adaptor sequences, generating clean reads. The clean reads were then aligned to the onion reference genome using HISAT2 (Version 2.2.1). Gene expression levels were quantified with featureCounts and normalized to FPKM values. DEGs were screened using the DESeq2 package with threshold set as |log_2_FC| ≥ 1 and FDR < 0.05. Finally, all identified DEGs were functionally annotated and subjected to enrichment analysis against GO, KEGG, and KOG databases.

### 4.8. Determination of Ascorbic Acid Content

Ascorbic acid was extracted from samples using 1.0% (*w*/*v*) oxalic acid according to the method described by Sun et al. [31]. The extract was centrifuged at 4000× *g* for 5 min and filtered through a 0.45 μm cellulose acetate filter. Separation was performed on a Waters Spherisorb C18 column (250 × 4.6 mm i.d., 5 μm particle size) using 0.1% oxalic acid as the mobile phase at a flow rate of 1.0 mL·min^−1^. Pure ascorbic acid was used as the standard, and total ascorbic acid content was quantified based on absorbance measured at 243 nm.

### 4.9. Determination of Total Phenol Content

Total phenols were extracted with 50% ethanol. The ethanolic extract was centrifuged at 4000× *g* for 5 min, and the supernatant was collected and incubated in the dark for 24 h. Subsequently, 0.2 mol·L^−1^ Folin–Ciocalteu reagent was added, followed by saturated sodium carbonate solution after 3 min. After standing at room temperature for 20 min, absorbance was determined at 760 nm using a spectrophotometer, with gallic acid as the standard [32].

### 4.10. FRAP Assay

The working FRAP reagent was prepared by mixing 300 mmol·L^−1^ acetate buffer (pH 3.6), 20 mmol·L^−1^ ferric chloride, and 10 mmol·L^−1^ 2,4,6-tripyridyl-s-triazine dissolved in 40 mmol·L^−1^ hydrochloric acid at a volume ratio of 10:1:1. Sample extracts were mixed with working FRAP reagent, vortexed, and incubated at 37 °C. Absorbance was measured at 593 nm after 10 min using a spectrophotometer [33].

### 4.11. ABTS Assay

Samples were extracted with 50% ethanol, and ABTS^+^ solution was added to the extract. After 2 h, absorbance was determined at 734 nm using a spectrophotometer [33].

### 4.12. Determination of Pigments Content

Anthocyanins were extracted with methanol-water-acetic acid solution (85:15:0.5, *v*/*v*/*v*), while chlorophylls and carotenoids were extracted with 90% acetone solution. Samples were vortexed, ultrasonic-treated, and extracted in darkness. HPLC analysis was performed using an Agilent 1260 system equipped with a VWD detector (Agilent Technologies, Santa Clara, CA, USA). For anthocyanins, separation was achieved using 5% aqueous formic acid and acetonitrile as mobile phases, with absorbance monitored at 530 nm. For chlorophylls and carotenoids, isopropanol and 80% aqueous acetonitrile were used as mobile phases, with detection wavelengths set at 448 nm and 428 nm, respectively [34].

### 4.13. Data Analysis

Statistical analyses were performed using Excel 2021, DPS 9.01, and GraphPad Prism 10.1.2. For bulb diameter, single bulb weight and moisture content, each individual bulb was regarded as one biological replicate, and each index was measured once per bulb. All results were expressed as mean ± standard deviation (SD) of three biological replicates. Significant differences among treatments were evaluated by one-way analysis of variance (ANOVA), followed by the least significant difference (LSD) test at the 0.05 probability level.

## 5. Conclusions

**Figure 9:**
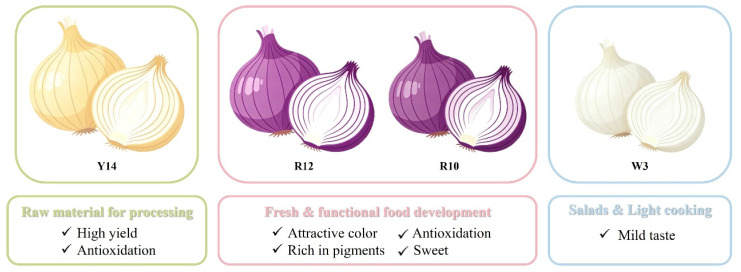
Schematic diagram of characteristics and utilization of four onion cultivars.

In conclusion, this study demonstrates that yellow, red, and white onions exhibit significant differences in phenotypic traits, metabolite profiles, volatile composition, antioxidant capacity, and flavor. Yellow onion Y14 shows high yield, strong antioxidant capacity, and moderate flavor, representing an ideal candidate for food processing. Red onion R12 features bright color and high carotenoid content, and is appropriate for both fresh eating and processing. Red onion R10 has the advantages of high contents of phenolics and anthocyanins, strong antioxidant activity, abundant volatile substances, and prominent sweet flavor, making it suitable for fresh consumption and functional food development. White onion W3 has tender flesh and mild taste, and is preferred for salads and light cooking (Figure 9). This multi-omics study uncovered the metabolic and transcriptional mechanisms underlying quality divergence in colored onions. On the basis of our findings, we put forward practical suggestions for onion breeding: key genes from phenylpropanoid, carotenoid and sugar metabolic pathways, including *DFR*, *PSY* and *PDS*, can be prioritized for molecular marker development and marker-assisted selection, to breed new varieties with ideal bulb color, high antioxidant activity and desirable flavor. For further research, we suggest carrying out functional characterization of candidate genes and exploring the regulatory relationships among metabolic pathways, which will further deepen our understanding of the mechanisms underlying onion quality formation. Collectively, this research provides solid theoretical support and excellent germplasm for the quality improvement and rational utilization of onion resources.

## 6. Limitations

This study did not perform independent qPCR validation to confirm the expression levels of core functional genes screened by transcriptomic analysis. Strict RNA integrity control, standardized transcriptome library sequencing, and consistent covariation among phenomic, metabolomic and transcriptomic datasets ensure the internal reliability of current omics results, whereas the lack of in vitro qPCR verification is an unavoidable limitation of this work. Subsequent trials with newly collected onion bulb samples will be arranged to complete targeted qPCR verification, so as to consolidate the regulatory mechanism underlying agronomic trait variation among different onion cultivars.

## Data Availability

The data presented in this study are available on request from the corresponding author. The data are not publicly available due to privacy and ethical restrictions.

## Supplementary Materials

The following supporting information can be downloaded at: https://www.mdpi.com/article/10.3390/plants15131949/s1, Figure S1: Metabolite profiling of four onion cultivars; Figure S2: Volatile metabolite and flavor analysis of four onion cultivars.
