# Supplementary material for: Metabolic and Transcriptomic Basis of Quality Divergence in Onions (Allium cepa L.) with Different Bulb Colors
Source: Plants (Basel). 2026 Jun 24;15(13):1949. doi: 10.3390/plants15131949 (PMC13363826; doi:10.3390/plants15131949)
Supplement: Supplementary file 1 [file plants-15-01949-s001.zip › plants-4384076-supplementary.pdf]

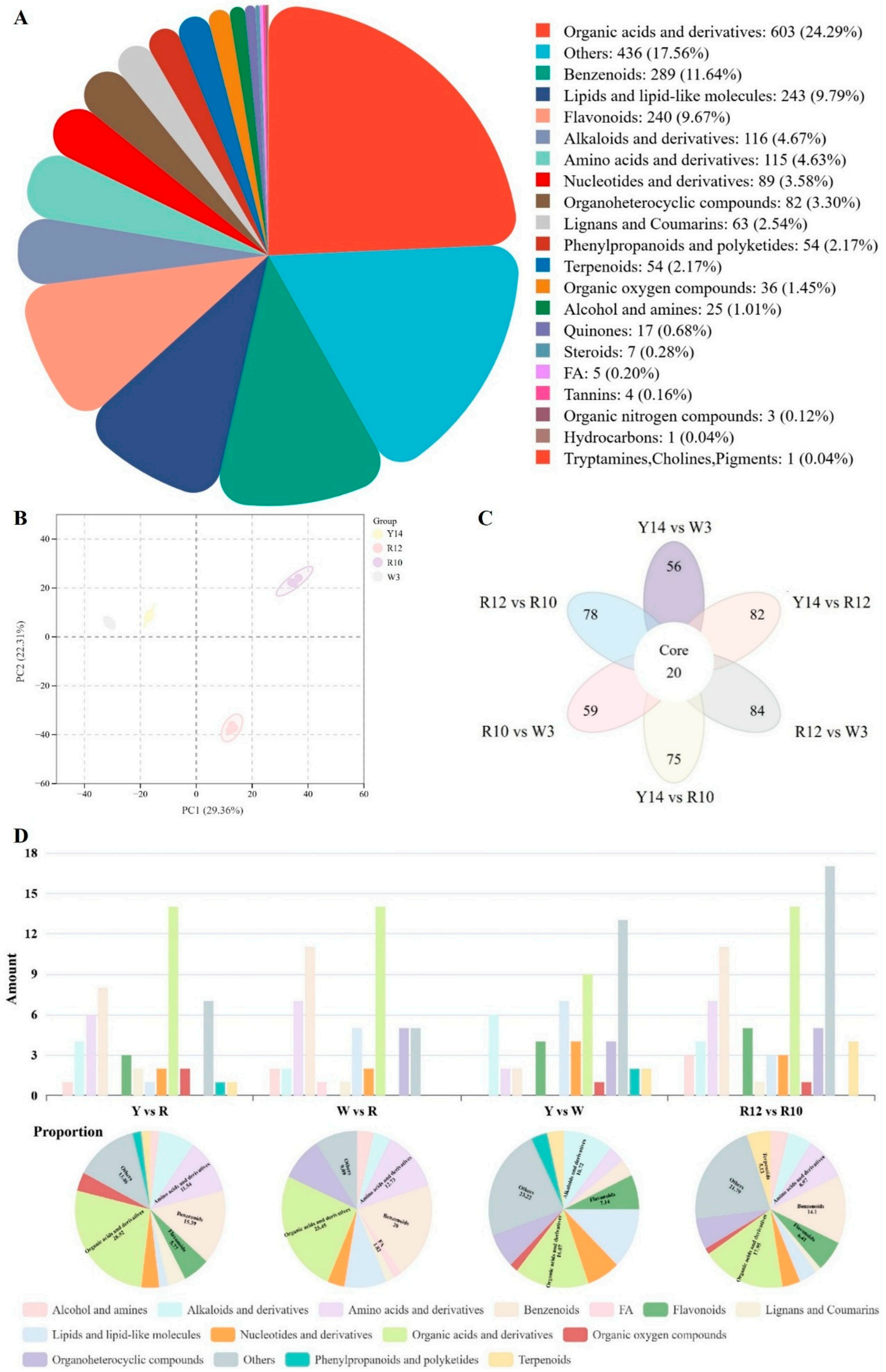

Figure S1 Metabolite profiling of four onion cultivars. (A) Classification of metabolites; (B) KEGG enrichment analysis of metabolites; (C) Venn diagram of differential groups;

(D) Types and proportion of specific differential metabolites among different comparison groups.

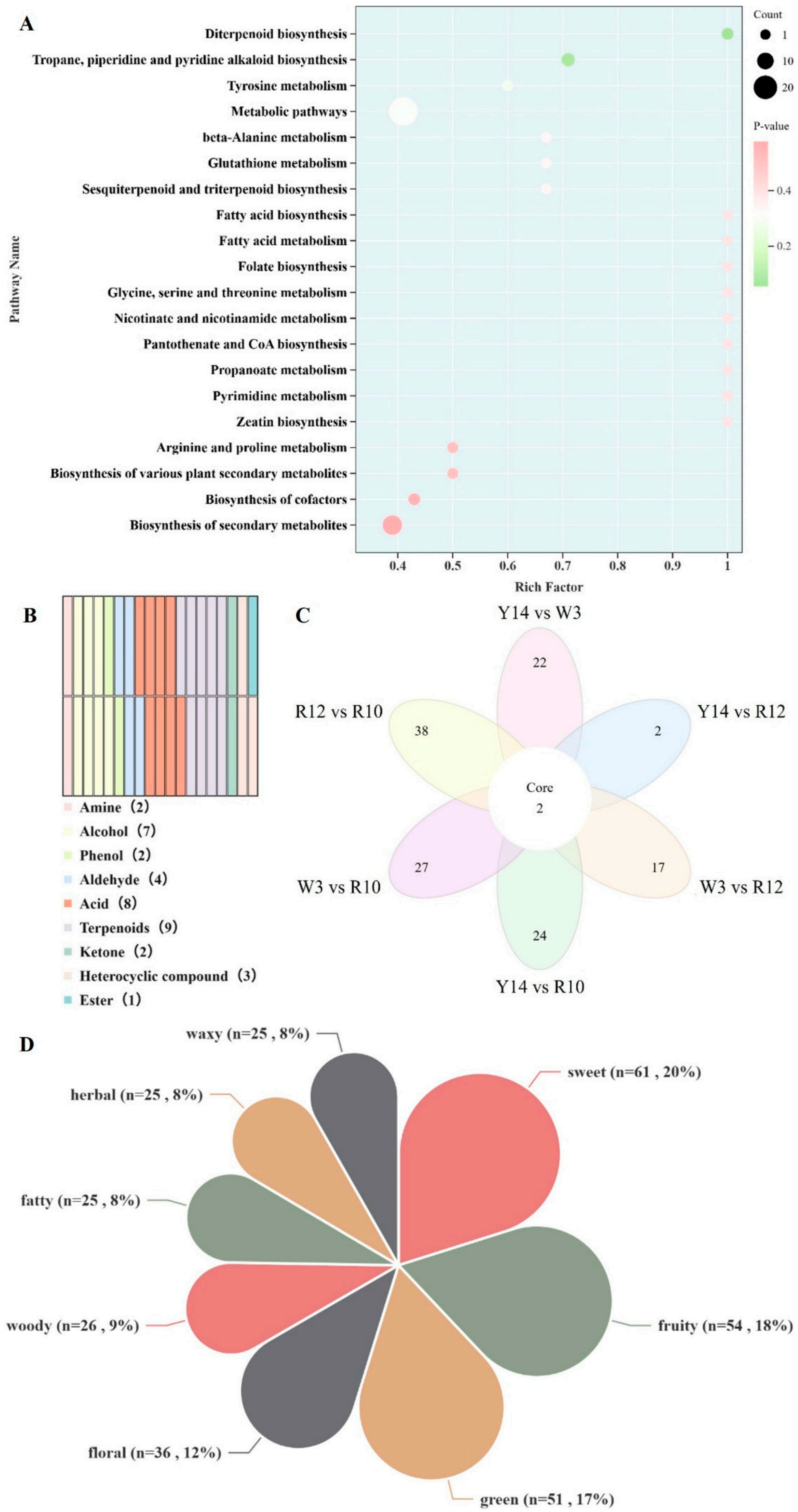
A
Diterpenoid biosynthesis
Tropane, piperidine and pyridine alkaloid biosynthesis
Tyrosine metabolism
Metabolic pathways
beta-Alanine metabolism
Glutathione metabolism
Sesquiterpenoid and triterpenoid biosynthesis
Fatty acid biosynthesis
Fatty acid metabolism
Folate biosynthesis
Glycine, serine and threonine metabolism
Nicotinate and nicotinamide metabolism
Pantothenate and CoA biosynthesis
Propanoate metabolism
Pyrimidine metabolism
Zeatin biosynthesis
Arginine and proline metabolism
Biosynthesis of various plant secondary metabolites
Biosynthesis of cofactors
Biosynthesis of secondary metabolites
Pathway Name
Rich Factor
0.4 0.5 0.6 0.7 0.8 0.9 1
Count
1
10
20
P-value
0.4
0.2
B
Amine（2）
Alcohol（7）
Phenol（2）
Aldehyde（4）
Acid（8）
Terpenoids（9）
Ketone（2）
Heterocyclic compound（3）
Ester（1）
C
Y14 vs W3
22
R12 vs R10
38
Y14 vs R12
2
Core
2
W3 vs R10
27
W3 vs R12
17
Y14 vs R10
24
D
waxy (n=25 , 8%)
sweet (n=61 , 20%)
herbal (n=25 , 8%)
fatty (n=25 , 8%)
woody (n=26 , 9%)
fruity (n=54 , 18%)
floral (n=36 , 12%)
green (n=51 , 17%)

Figure S2 Volatile metabolite and flavor analysis of four onion cultivars. (A) KEGG enrichment analysis of volatile differential metabolites; (B) Classification of volatile differential metabolites; (C) Venn diagram of volatile differential metabolites in different groups; (D) Classification of flavor-related metabolites.
